# COVID-19 vaccination influences subtypes of γδ-T cells during pregnancy

**DOI:** 10.3389/fimmu.2022.900556

**Published:** 2022-10-12

**Authors:** Li Wang, Jiawei Li, Silin Jiang, Yan Li, Rong Guo, Yuyuan Chen, Yan Chen, Hang Yu, Qingqing Qiao, Mingjie Zhan, Zhinan Yin, Zheng Xiang, Chengfang Xu, Yan Xu

**Affiliations:** ^1^ The Biomedical Translational Research Institute, Faculty of Medical Science, Jinan University, Guangzhou, China; ^2^ Department of Obstetrics and Gynecology, The Third Affiliated Hospital of Sun Yat-Sen University, Guangzhou, China; ^3^ Guangzhou Purui Biotechnology Co., Ltd., Guangzhou, China; ^4^ National Center for International Research of Bio-targeting Theranostics, Guangxi Key Laboratory of Bio-targeting Theranostics, Collaborative Innovation Center for Targeting Tumor Diagnosis and Therapy, Guangxi Talent Highland of Bio-targeting Theranostics, Guangxi Medical University, Nanning, China; ^5^ Department of Obstetrics and Gynecology, The Second Affiliated Hospital of Soochow University, Suzhou, China; ^6^ Department of Obstetrics and Gynecology, The First Affiliated Hospital of Gannan Medical University, Ganzhou, China; ^7^ Guangdong Provincial Key Laboratory of Tumor Interventional Diagnosis and Treatment, Zhuhai Institute of Translational Medicine Zhuhai People’s Hospital Affiliated with Jinan University, Jinan University, Zhuhai, China

**Keywords:** COVID-19, vaccination, pregnancy, γδ-T cells, Vδ1^+^ T cells, Vδ2^+^ T cells

## Abstract

Up to now, there has been insufficient clinical data to support the safety and effects of vaccination on pregnancy post COVID-19 vaccination. The γδ-T cells are considered an important component in the immune system to fight against viral infection and exhibit critical roles throughout the pregnancy period. However, the immunological roles of γδ-T cells in pregnant women with the COVID-19 vaccination remain unclear. Therefore, the objective of this study is to investigate the alteration of frequency and expression pattern of activation receptors and inhibitory receptors in γδ-T cell and its subsets in peripheral blood samples collected from non-pregnant vaccinated women, vaccinated pregnant women, and unvaccinated pregnant women. Our findings indicated that the frequency of CD3^+^γδ-T^+^ cells is lower in vaccinated pregnant women than in unvaccinated pregnant women. But no significant difference was found in the frequency of CD3^+^γδ-T^+^ cells between non-pregnant vaccinated women and vaccinated pregnant women. In addition, there were no significant differences in the frequencies of CD3^+^γδ-T^+^Vδ1^+^T cells, CD3^+^γδ-T^+^Vδ2^+^T cells, CD3^+^γδ-T^+^Vδ1^-^Vδ2^-^T cells, and Vδ1^+^T cell/Vδ2^+^T cell ratio between the pregnant women with or without COVID-19 vaccination. Similar results were found after comparing non-pregnant and pregnant women who received the COVID-19 vaccine. However, there was a significant difference in the fraction of Vδ1^-^Vδ2^-^T cells in CD3^+^γδ-T^+^ cells between non-pregnant vaccinated women and vaccinated pregnant women. The frequency of NKG2D^+^ cells in Vδ2^+^T cells was not significantly different in the vaccinated pregnant women when compared to that in unvaccinated pregnant women or non-pregnant vaccinated women. But the percentage of NKG2D^+^ cells in Vδ1^+^T cells was the lowest in pregnant women after COVID-19 vaccination. Furthermore, down-regulation of NKP46 and NKP30 were found in Vδ2^+^T and Vδ1^+^T cells in the vaccinated pregnant women, respectively. After the vaccination, up-regulation of PD-1 expression in Vδ1^+^T cells and Vδ2^+^T cells indicated γδ-T cells could respond to COVID-19 vaccination and display an exhausted phenotype following activation. In conclusion, COVID-19 vaccination influences subtypes of γδ-T cells during pregnancy, but the side effects might be limited. The phenotypical changes of Vδ1^+^T cells and Vδ2^+^T cells will be a promising predictor for evaluating the clinical outcome of the COVID-19 vaccine.

## Introduction

Due to a lack of clinical and scientific knowledge, the challenge of the Coronavirus Disease 2019 (COVID-19) pandemic has exposed the limitations of our understanding about severe acute respiratory syndrome coronavirus 2 (SARS-CoV-2) as well as the immune response during viral infection and vaccination (1). Pregnant women especially are at increased risk of severe illness from COVID-19. There have been a few studies that demonstrated the efficacy and safety of COVID-19 vaccines in pregnant women, who have been excluded from the clinical trials because of the ethical issues (2). However, we still struggle with a complete understanding about whether vaccination could modulate the immune response of pregnant women (3). During normal human pregnancy, γδ-T cells have been reported to play a role in this process (4).

Human γδ T cells can be divided into three subgroups, Vδ1, Vδ2 and Vδ3, according to the structural differences of γ and δ chains. Although γδ-T cells represent only a small fraction of T lymphocytes (1-10%) (5), they have a variety of immune functions, such as resistance to virus infection (6), inflammatory regulation, tissue homeostasis (6), helping B cells to produce antibodies (7), and even maintenance of successful pregnancy (6). Some studies reported that, during normal human pregnancy, γδ-T cells secrete some anti-inflammatory cytokines to reduce nature killer activity (8, 9).Indeed, γδ-T cells regulate the release of inflammatory factors such as IFN-γ,TNF-α, granzyme A/B, and perforin by regulating the expression of surface-activated receptors such as NKG2D, NKp30, NKP46, and the inhibitory receptor PD-1 to regulate their cytotoxic functions (10–13). In addition, low cytotoxic activity of γδ-T cells is necessary during normal pregnancy (14). Most importantly, the imbalance between Vδ1^+^ T cells and Vδ2^+^ T cells was observed in adverse pregnancy (15).

Therefore, in this study, we aimed to interrogate the alternation of subtypes of γδ-T cells in pregnant women after COVID-19 vaccination, which could provide a better understanding of the role of γδ-T cells in pregnant women after COVID-19 vaccination, and in turn can further help us to monitor and evaluate the safety and efficacy of COVID-19 vaccines during the pregnancy period.

## Materials and methods

### Study population

The research objects were selected from pregnant women vaccinated against COVID-19 who went to Tianhe Campus of the Third Affiliated Hospital of Sun Yat-sen University in Guangzhou, China from August 2021 to February 2022. These women did not confirm their pregnancies until after they had received the COVID-19 vaccine. The inclusion criteria included: (1) age of 18-35 years; (2) singleton pregnancy; (3) the onset of pregnancy as calculated by crown-rump length (CRL) on NT ultrasound, with at least one dose of vaccine after pregnancy; and (4) signing an informed consent form, providing vaccination information and confirming their participation in the study. The exclusion criteria included: (1) the gestational age of the documented prenatal examination is not 11-13+6 weeks; (2) other vaccines have been received within 1 year, such as HPV vaccine and hepatitis B vaccine; or (3) complications with basic diseases, requiring long-term medication. The peripheral blood of the patients enrolled in this study was collected to perform immunological assays. The study included 27 vaccinated pregnant women, 11 unvaccinated pregnant women, and 20 non-pregnant vaccinated women. The clinical and demographic characteristics and vaccination scheme of pregnant women were described in **Tables 1**, **2**, respectively. This study was approved by the Ethics Committee of the Third Affiliated Hospital of Sun Yat-sen University in Guangzhou.

**Table 1:** Basic characteristics of vaccinated and unvaccinated pregnant women.

Groups	Non-pregnant vaccinated group	Un-vaccinated group	Vaccinated group
Included	20	11	27
Age (median and range)	24 (23-35)	29 (26-33)	29 (28-32)
Previous history of SARS-CoV-2 infection	No	No	No
Hypertension	No	No	No
Diabetes	No	No	No
Gestational age of laboratory tests (weeks, median and range)	No	12.4 (12.3-12.7)	12.9 (12.6-13.3)
NT (mm, mean and standard deviation)	No	1.4 (0.4)	1.4 (0.4)
Gestational age of laboratory tests (weeks, median and range)	No	12.4 (12.3-12.7)	12.9 (12.6-13.3)

Gestational age is equal to the number of gestational days divided by 7.

**Table 2:** Scheme of vaccination of vaccinated pregnant women.

Vaccinated pregnant women	Pregnancy time	Date of laboratory tests	Scheme of vaccination	
			Date of 1st dose	Date of 2nd dose
Vg-1	2021/05/30	2021/08/27	2021/07/27	–
Vg-2	2021/06/03	2021/08/27	2021/06/12	–
Vg-3	2021/05/31	2021/08/27	2021/05/22	2021/06/29
Vg-4	2021/05/25	2021/08/30	2021/05/23	2021/06/22
Vg-5	2021/06/08	2021/08/30	2021/06/13	2021/04/30
Vg-6	2021/05/30	2021/08/30	2021/06/10	2021/07/13
Vg-7	2021/05/29	2021/08/30	2021/06/23	–
Vg-8	2021/05/29	2021/08/30	2021/06/02	2021/05/04
Vg-9	2021/06/04	2021/09/01	2021/07/01	–
Vg-10	2021/05/31	2021/09/01	2021/04/14	2021/06/07
Vg-11	2021/06/04	2021/09/02	2021/05/29	2021/06/26
Vg-12	2021/06/06	2021/09/06	2021/05/10	2021/06/09
Vg-13	2021/06/13	2021/09/10	2021/05/06	2021/06/16
Vg-14	2021/06/06	2021/09/10	2021/05/09	2021/06/12
Vg-15	2021/06/16	2021/09/15	2021/07/16	–
Vg-16	2021/06/25	2021/09/18	2021/05/29	2021/06/25
Vg-17	2021/06/26	2021/09/22	2021/04/22	2021/07/08
Vg-18	2021/06/28	2021/09/26	2021/06/23	2021/07/19
Vg-19	2021/06/26	2021/09/27	2021/06/28	2021/07/20
Vg-20	2021/06/26	2021/09/27	2021/05/30	2021/06/26
Vg-21	2021/06/24	2021/09/27	2021/05/29	2021/06/29
Vg-22	2021/07/15	2021/10/12	2021/07/11	2021/08/07
Vg-23	2021/07/24	2021/10/12	2021/07/25	2021/08/24
Vg-24	2021/07/06	2021/10/11	2021/07/19	–
Vg-25	2021/07/23	2021/10/14	2021/06/29	2021/07/21
Vg-26	2021/07/20	2021/10/15	2021/06/20	2021/07/28
Vg-27	2021/07/17	2021/10/19	2021/07/20	–
Np-1	–	–	2021/04/22	2021/05/27
Np-2	–	–	2021/04/22	2021/05/25
Np-3	–	–	2021/04/01	2021/05/25
Np-4	–	–	2021/04/08	2021/05/07
Np-5	–	–	2021/04/24	2021/05/11
Np-6	–	–	2021/04/02	2021/05/31
Np-7	–	–	2021/04/02	2021/04/30
Np-8	–	–	2021/04/02	2021/04/30
Np-9	–	–	2021/04/02	2021/04/30
Np-10	–	–	2021/04/02	2021/04/30
Np-11	–	–	2021/04/02	2021/04/30
Np-12	–	–	2021/04/02	2021/04/30
Np-13	–	–	2021/04/02	2021/04/30
Np-14	–	–	2021/04/02	2021/04/30
Np-15	–	–	2021/04/02	2021/04/30
Np-16	–	–	2021/03/11	2021/04/12
Np-17	–	–	2021/03/11	2021/04/12
Np-18	–	–	2021/03/11	2021/04/12
Np-19	–	–	2021/03/11	2021/04/12
Np-20	–	–	2021/03/11	2021/04/12

### Immune cell phenotype analyzing by flow cytometry

Collected peripheral blood samples were analyzed using flow cytometry. Peripheral blood mononuclear cells (PBMCs) were isolated using Ficoll-Paque centrifugation, then stained by the following antibodies: anti-human CD3-APC-H7 (BD biosciences, clone: SK7), anti-human TCR γδ-BV421 (BD biosciences, clone: 11F2), anti-human PD-1-BB515 (BD biosciences, clone: EH12.1), anti-human NKP46-BV510 (BD biosciences, clone: 9E2/NKP46), anti-human NKP30-Alexa Fluor^®^647 (BD biosciences, clone: P30-15), anti-human NKG2D-PE-Cy™7 (BD biosciences, clone: 1D11), anti-human TCR Vδ2-PE (BD biosciences, clone: B6), and anti-human TCR Vδ1-PerCP-Vio700 (Miltenyi Biotec, clone: REA173). Data was analyzed using FlowJo 10.1 software (Tree Star Inc., Ashland, OR, USA).

### Statistical analysis

Statistical analyses were performed using GraphPad Prism (GraphPad Software, Inc.). All results are expressed as the mean ± SEM (standard error of the mean). To analyze the difference in γδ T cells and its subsets between vaccinated women and unvaccinated women, Mann-Whitney U tests were performed.

## Results

### The frequencies of γδ T cell and its subsets in pregnant women after COVID-19 vaccination

**Figure 1:**
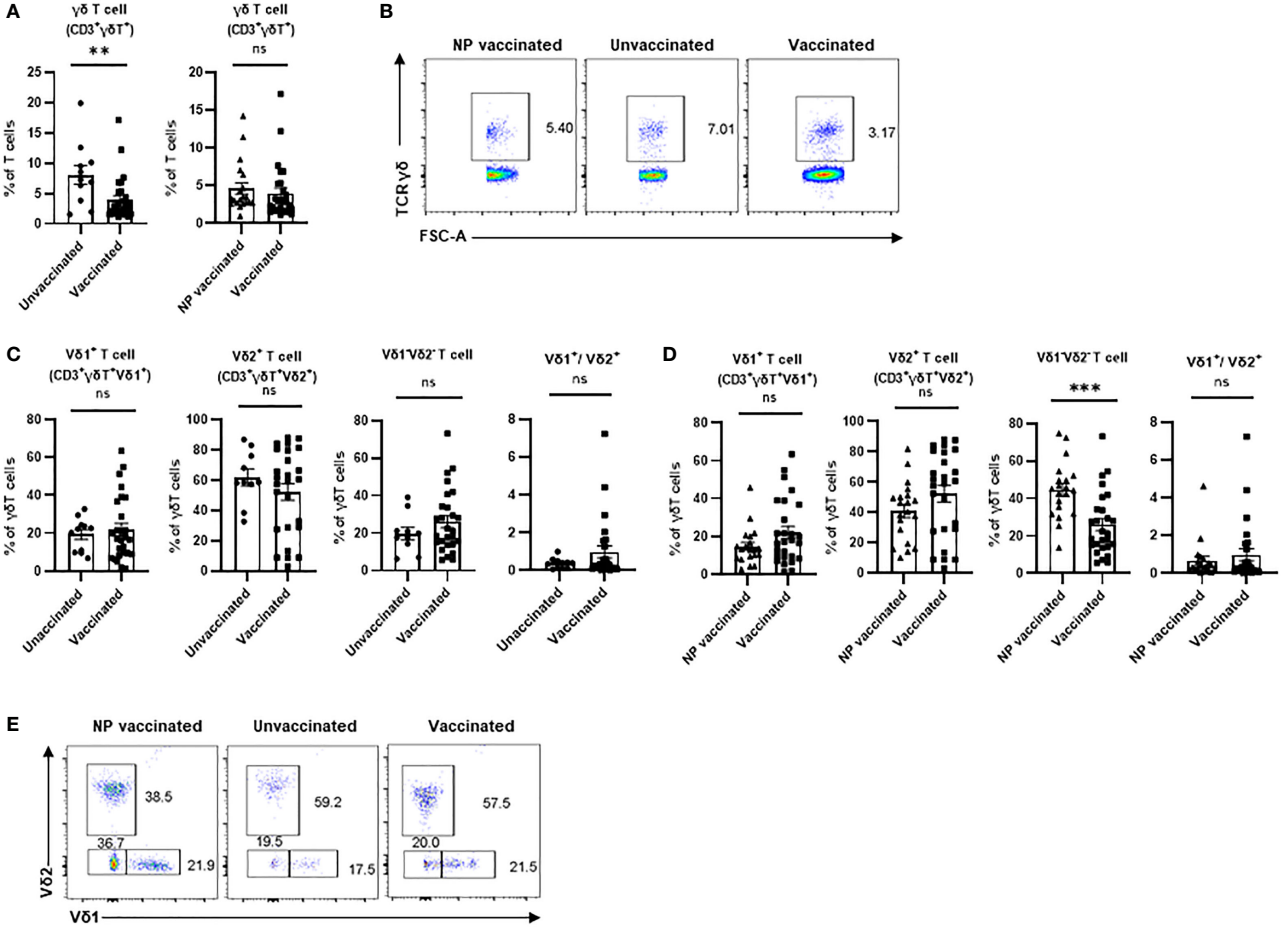
Peripheral γδ T cells and their subsets in non-pregnant vaccinated women, vaccinated, and unvaccinated pregnant women. Statistical comparison of γδ T cell proportions in CD3^+^ T cells **(A, B)** Typical flow cytometry plots and gating for a CD3^+^TCR γδ^+^;Vδ1^+^, Vδ2^+^, and Vδ1^-^Vδ2^-^ subsets in γδ T cells **(C, D)**, and the Vδ1^+^/Vδ2^+^ ratio between the vaccinated and unvaccinated pregnant women. **(E)** Gate from TCR γδ^+^ cells,Vδ1^+^, Vδ2^+^, and Vδ1^-^Vδ2^-^ subsets are shown as representative flow cytometry plots. ns, no significance; **P < 0.01. ***P < 0.001. (NP vaccinated, non-pregnant vaccinated).

The aim of this study was to investigate whether COVID-19 vaccination influences the distribution of total γδ T cells, Vδ1^+^T cells, Vδ2^+^T cells, and Vδ1^-^Vδ2^-^T cells in the peripheral blood samples of 58 individuals enrolled in this study. Total γδ T cell was identified among CD3^+^ lymphocytes using flow cytometry (**Figures 1A, B**). The results indicated that frequency of total γδ T cells was markedly higher in unvaccinated pregnant women compared to vaccinated pregnant women, but there was no significant difference between non-pregnant vaccinated women and vaccinated pregnant women. Furthermore, to investigate the impact of COVID-19 vaccination on frequencies of γδ T cells subsets, we next compared Vδ2^+^T cells, Vδ1^+^T cells, and Vδ1^-^Vδ2^-^ T cells’ frequencies as well as Vδ1/Vδ2 ratio in non-pregnant vaccinated women, vaccinated pregnant women, and unvaccinated pregnant women. The results indicated that there were no significant differences in frequencies of Vδ1^+^T, Vδ2^+^T cells, and Vδ1^-^Vδ2^-^ T cells in CD3^+^γδ^+^ T cells between vaccinated pregnant women and unvaccinated pregnant women (**Figures 1C, E**). However, compared with non-pregnant vaccinated women, the proportion of Vδ1^-^Vδ2^-^ T cells in CD3^+^γδ^+^ T was significantly reduced in vaccinated pregnant women (**Figure 1D**). Additionally, Vδ1/Vδ2 ratio was similar in these women with or without vaccination (**Figure 1D**).

### Phenotypical changes of Vδ2^+^T cells in vaccinated pregnant women

**Figure 2:**
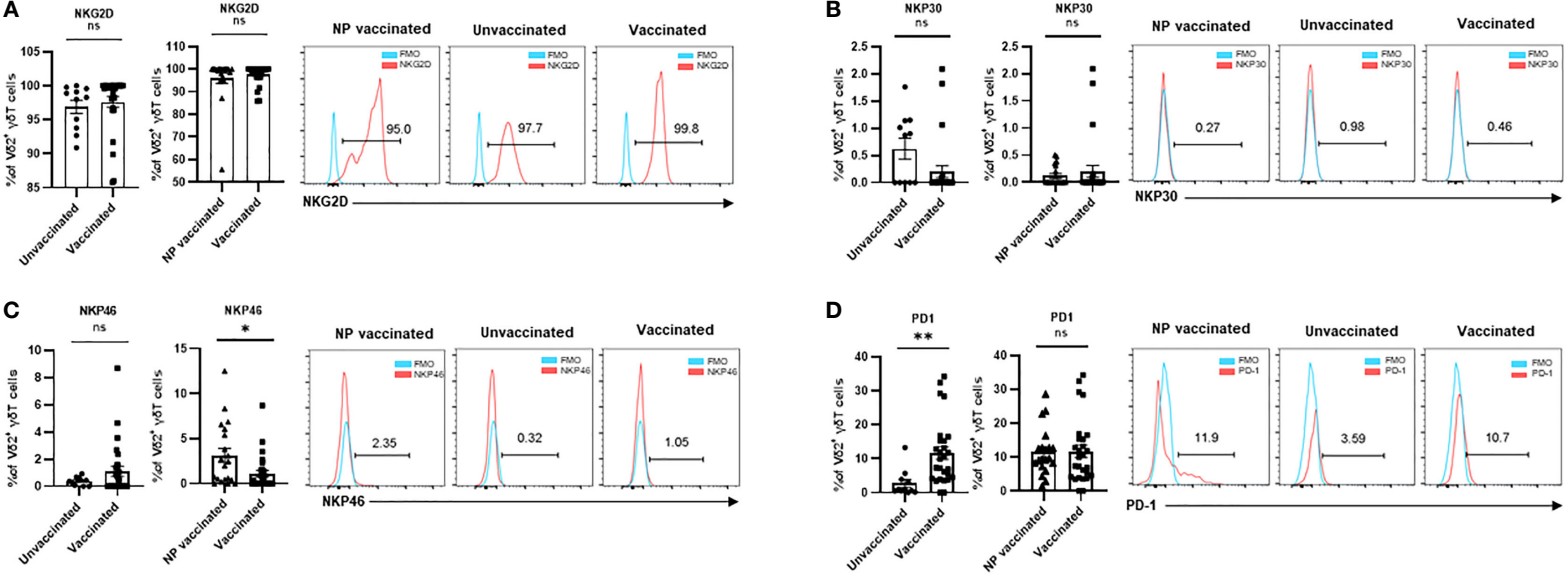
Expressions of crucial molecular of Vδ2^+^ γδ T cells in non-pregnant vaccinated women, vaccinated, and unvaccinated pregnant women. Comparable analysis of expressions of NKG2D **(A)**, NKP30 **(B)**,NKP46 **(C)** and PD-1 **(D)** receptors of Vδ2^+^ γδ T cells acquired by flow cytometry. ns, no significance; *P < 0.05, **P < 0.01.

It is well known that the activating and inhibitory receptors define the degree of immune cell maturation and responsiveness to stimuli, so we next investigated the frequencies of NKG2D^+^, NKp30^+^, NKp46^+^, and PD-1^+^ cells in Vδ2^+^T cells. We found no significant difference in the proportions of NKG2D^+^ Vδ2^+^T cells among these three groups (**Figure 2A**). γδ T cell subsets usually express activating natural killer (NK) receptors, such as NKp30 and NKp46, which are involved in regulating immunological functions of γδ T cell and its subsets. There was no significant difference in percentage of NKp30^+^Vδ2^+^T cells between vaccinated pregnant women and unvaccinated pregnant women (**Figure 2B**). In addition, a similar result was found in comparison of vaccinated pregnant women and non-pregnant vaccinated women (**Figure 2B**). By contrast, there was no significant difference in the percentage of NKp46^+^Vδ2^+^T cells between vaccinated pregnant women and unvaccinated pregnant women. But compared with non-pregnant vaccinated women, the percentage of NKp46^+^Vδ2^+^T cells was significantly decreased in vaccinated pregnant women (**Figure 2C**). Furthermore, we supposed that γδ T cell would exhibit the exhausted phenotype after vaccination. Actually, the frequency of PD-1^+^ Vδ2^+^T cells was significantly elevated in vaccinated pregnant women compared to unvaccinated pregnant women. However, there was no difference between non-pregnant vaccinated women and vaccinated pregnant women (**Figure 2D**).

### Phenotypical changes of Vδ1^+^T cells in vaccinated pregnant women

**Figure 3:**
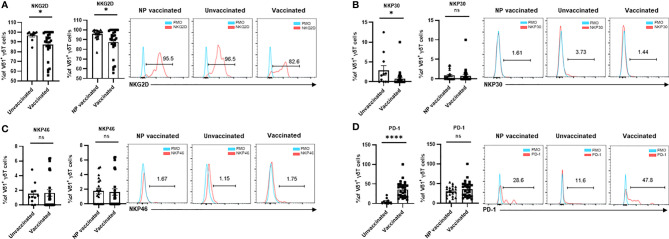
Expressions of crucial molecular of Vδ1^+^ γδ T cells in non-pregnant vaccinated women, vaccinated, and unvaccinated pregnant women. Comparable analysis of expressions of NKG2D **(A)**, NKP30 **(B)**,NKP4 6 **(C)**, and PD-1 **(D)** receptors of Vδ1^+^ γδ T cells acquired by flow cytometry. ns, no significance; *P < 0.01; ****P < 0.001.

To investigate the alteration of expression pattern of activating and inhibitory receptors in Vδ1^+^T cells after COVID-19 vaccination in the pregnant women, the frequencies of NKG2D^+^, NKp30^+^, NKp46^+^, and PD-1^+^ cells in Vδ2^+^T cells were determined. Unsimilar to Vδ2^+^T cells, the frequencies of NKG2D^+^ and NKp30^+^ cells in Vδ1^+^ cells were much lower in vaccinated pregnant women than that in unvaccinated pregnant women (**Figures 3A, B**). In addition, a significant difference in the frequency of NKG2D^+^Vδ1^+^ cells was found between non-pregnant vaccinated women and vaccinated pregnant women. Similar to Vδ2^+^T cells, the frequency of NKP46^+^ cells were no different in all three groups (**Figure 3C**). Finally, PD-1^+^ Vδ1^+^T cells were significantly increased in the pregnant women after vaccination (**Figure 3D**).

## Discussion

Our study demonstrated that frequencies of total γδ T cell (CD3^+^γδ^+^ T cells) was significantly decreased in the pregnant women with COVID-19 vaccination compared to that in the pregnant women without COVID-19 vaccination. Furthermore, analysis of the frequencies of Vδ1^+^T cells and Vδ2^+^T cells in total γδ T cells indicated that there were no significant differences between vaccinated pregnant women and unvaccinated pregnant women. Importantly, no significant difference was found on the Vδ1/Vδ2 ratio between these two groups of women who were with or without COVID-19 vaccination. For evaluation of activated and exhausted phenotypes in of Vδ1^+^T cells and Vδ2^+^T cells after COVID-19 vaccination in the pregnant women, the frequencies of NKG2D^+^, NKp30^+^, NKp46^+^, and PD-1^+^ cells in these two subsets were analyzed. Our findings suggested that Vδ1^+^T cells and Vδ2^+^T cells developed an exhausted phenotype post COVID-19 vaccination. These results demonstrate that COVID-19 vaccination exhibits a certain degree of influence on the frequency of total γδ T cell and alteration of phenotype of Vδ1^+^T cells and Vδ2^+^T cells. However, the Vδ1/Vδ2 ratio is similar between vaccinated pregnant women and unvaccinated pregnant women, which indicates that vaccination did not break the important balance between these two main subsets of peripheral γδ T cells. Taken together, the results suggest COVID-19 vaccination influences subtypes of γδ-T cells without effects on pregnancy.

In this study, we found that the frequency of total γδ-T cells was similar between the pregnant women and non-pregnant women after COVID-19 vaccination (**Figure 1A**). In addition, there were no significant differences in frequencies of Vδ1^+^T cells and Vδ2^+^T cells between these two groups of individuals. A similar result was also found in the Vδ1^+^T/Vδ2^+^T ratio (**Figure 1C**). However, the frequency of Vδ1^-^Vδ2^-^ T cells was much higher in the non-pregnant vaccinated women compared with that in vaccinated pregnant women (**Figure 1D**). Furthermore, our data indicated that the proportions of NKP46^+^Vδ2^+^T cells and NKG2D^+^Vδ1^+^T cells were lower in the vaccinated pregnant women than that in non-pregnant vaccinated women (**Figures 2C**, **3A**). By contrast, no significant differences were found in the percentages of NKG2D^+^, NKP30^+^, and PD-1^+^ cells in Vδ2^+^T cells and NKP30^+^ and NKP46^+^ cells in Vδ1^+^T cells between the non-pregnant vaccinated women and vaccinated pregnant women (**Figures 2A, B, D**; **Figures 3B-D**). These data further demonstrated that pregnancy is not a potential influencing factor for our findings, and this will help us to achieve more confident conclusions.

In humans, Vδ1^+^T cells and Vδ2^+^T cells are the two major subsets of γδ T cells which are identified by the Vδ chains. Vδ1^+^T cells constitute the majority of T cells in the thymus and mucosal tissues, and Vδ2^+^T cells are predominant in the peripheral blood (16). As the main γδ T cell subsets, Vδ1^+^T cells and Vδ2^+^T cells exhibit different immunological functions. Vδ1^+^T cells display regulatory and effector features, and Vδ2^+^T cells exert a cytotoxic activity targeting pathogenic characteristics. According to previous studies, the frequency of peripheral γδ T cells is higher in women with a successful pregnancy compared to women with pregnancy failure (9). In addition, one study suggested that Vδ1^+^T cells could produce IL-10 to down-regulate the cytotoxic NK cells during pregnancy. In healthy pregnant women, the predominant subpopulation of peripheral γδ T cells is Vδ1^+^T cells, whereas Vδ2^+^T cells is the most frequent subset in women with recurrent miscarriage (16). Therefore, this evidence demonstrated that an imbalance of Vδ1/Vδ2 ratio leads to adverse pregnancy outcome (15, 16). It has been reported that γδ T cells (mostly expressing Vδ2) are able to destruct influenza A virus-infected cells as efficient as CD8^+^ T cells or NK cells in a polycytotoxic manner and by releasing IFN-γ against infected cells *in vitro* (17). In several contexts, including infection with Mtb, malaria, influenza, and HIV and vaccination with BCG and live attenuated influenza, there are clear patterns of γδ T-cell expansion, particularly of the Vδ2^+^ subset, in response to both infection and vaccination (18). However, there is no literature report on the effect of other vaccines after pregnancy on γδ T cells’ function. In the future, we could detect the difference in γδ T cells’ function between other vaccines and the COVID-19 vaccine during pregnancy, so as to explore whether this result is specific to the COVID-19 vaccine. In our study, COVID-19 vaccination did not change Vδ1/Vδ2 ratio in the pregnant women, which indicates the impact of vaccination might not cause an adverse outcome.

NK cell activating receptors, such as NKG2D, NKp30, and NKp46, are widely involved in regulating NK functions during pregnancy (19). Additionally, one recent study suggested that the frequency of NKG2D^+^Vδ2^+^T cells was negatively correlated with a successful clinical pregnancy (8). Our data demonstrate that no significant difference was found on the percentages of NKG2D^+^ cells in Vδ1^+^T cells and Vδ2^+^T cells, which suggest that COVID-19 vaccination did not induce the highly activated peripheral γδ T cells in the pregnant women. Because of the activated γδ T cells, a high level of inflammation is considered as the major cause of abortion (15). Additionally, frequencies of NKp30^+^ and NKp46^+^ cells in Vδ1^+^T and Vδ2^+^T were largely similar between vaccinated pregnant women and unvaccinated pregnant women. In summary, these data suggested that the immunological functions of γδ T cells were not altered after COVID-19 vaccination in the pregnant women.

Accordingly, many previous studies suggested that exhaustion of γδ T cells is accompanied by a decrease in the frequency of cells in different types of disease. For instance, in acute myeloid leukemia (AML), the data demonstrated that the proportion of total γδ T cells was decreased in AML patients (20). Subsequently, in these γδ T cells, the authors observed increased PD-1 expression and decreased NKG2D expression, indicating highly activated or even exhausted states in the γδ T cells at diagnosis of AML (20). Additionally, in the acute viral infection, a lower frequency of Vδ2+T cells was also observed (21). Moreover, these Vδ2^+^T cells highly expressed CD95, which in turn could lead to cell apoptosis that induces the loss of cells (21). Interestingly, this work demonstrated that the expression level of CTLA-4 exhaustion marker was elevated during acute viral infection. In line with these previous results, we also found that stimulation of COVID-19 vaccine induces the exhaustion status within the loss of γδ T cells in pregnant women, suggesting the involvement of γδ T cells in the complex network of protective response induced by COVID-19 vaccination. PD-1 expression is induced on activated T cells and is correlated to exhaustion status in anti-infection and anti-tumor responses (22, 23). In our study, we found that the frequency of PD-1^+^ cells in Vδ1^+^T and Vδ2^+^T were much higher in vaccinated pregnant women compared to unvaccinated pregnant women. We suspect that γδ T cells developed an exhausted phenotype following activation by COVID-19 vaccination. This switch from activation and exhaustion might be the reason why the frequency of γδ T cells was decreased in the pregnant women with COVID-19 vaccination. Since the objective of this study is to investigate whether COVID-19 vaccination influences subtypes of γδ-T cells during pregnancy, the changes of frequency and immunological phenotypes of γδ-T cells were determined in this study. γδ-T cells are well known as having multiple functions in innate immune cells, suggesting they play important roles in anti-viral infection and immune response to vaccination. The results of our study could be considered as the clues to address the questions about the potential immunological functions of γδ-T cell and its subsets involved in the immune activity associated with COVID-19 vaccination. This deserves further study in the future.

Despite the positives provided by this study, there are still some limitations. First, a further larger sample size study is warranted to validate these findings. And the effects of age, vaccination scheme, vaccine types, and other factors in impacting the immunological features of γδ T cell should be further evaluated in pregnant women after COVID-19 vaccination. Second, in this study, we only determined the phenotypic changes of γδ T cell subsets in the peripheral blood of individuals. The functional capacity of γδ T cell and its subpopulations are rarely investigated in COVID-19 vaccination and should be determined in the future. Third, we found the phenotypic changes of γδ T cell subsets by comparing the data collected in the pregnant women with or without COVID-19 vaccination. In the future, using samples from each vaccinated individual at different time points in the vaccination scheme will further help us to clarify the significance of functional alterations of γδ T cell and its subsets in establishing protective immunity against COVID-19 infection after vaccination.

Taken together, our study suggests that γδ T cell and its subsets could respond to COVID-19 vaccination and display an exhausted phenotype following activation. In addition, COVID-19 vaccination influences subtypes of γδ-T cells during pregnancy, but the side effects are limited. Last but not least, the contribution of γδ T cell and its subsets to the immunology of COVID-19 vaccination needs to be further investigated.

## Data availability statements

The raw data supporting the conclusions of this article will be made available by the authors, without undue reservation.

## Ethics statement

This study was approved by the Ethics Committee of the Third Affiliated Hospital of Sun Yat-sen University in Guangzhou, PR China. The participants provided their written informed consent to participate in this study. The patients/participants provided their written informed consent to participate in this study.

## Author contributions

CX and ZY designed the study; YX and ZX designed the study, performed the data analysis, and wrote the manuscript. LW and SJ enrolled the subjects and collected the peripheral blood, performed the experiments, and assisted in the preparation of the figures. JL, YL, and RG performed the data analysis and plotted the graphs. YYC, YC, HY, QQ, and MZ collected the peripheral blood and performed the experiments. All authors contributed to the article and approved the submitted version.

## Funding

This work was supported by the National Natural Science Foundation of China (82070606) and the Natural Science Foundation of Guangdong Province, China (2021A1515011441). This work was also supported by the National Natural Science Foundation of China (32000616 and 31570898) and the Basic and Applied Basic Research Fund of Guangdong Province (2020A1515111203 and 2016A030313112).

## Acknowledgments

We thank all participants and investigators involved in the study.

## Conflict of interest

Author JL was employed by the company Guangzhou PuruiBiotechnology Co., Ltd, Guangdong, China.

The remaining authors declare that the research was conducted in the absence of any commercial or financial relationships that could be construed as a potential conflict of interest.

## Publisher’s note

All claims expressed in this article are solely those of the authors and do not necessarily represent those of their affiliated organizations, or those of the publisher, the editors and the reviewers. Any product that may be evaluated in this article, or claim that may be made by its manufacturer, is not guaranteed or endorsed by the publisher.
